# Supplementary material for: Getting More Out of Biomedical Documents with GATE's Full Lifecycle Open Source Text Analytics
Source: PLoS Comput Biol. 2013 Feb 7;9(2):e1002854. doi: 10.1371/journal.pcbi.1002854 (PMC3567135; doi:10.1371/journal.pcbi.1002854)
Supplement: Dataset S1 — GATE software. Dataset S1 bundled with this paper contains a distribution of GATE (or see http://gate.ac.uk/download/). (TGZ) [file pcbi.1002854.s001.tgz › plos-gate/doc/plugins.html]

List of plugins available to GATE


  


Plugins for GATE

This page lists some of the plugins that are currently available with GATE:

- Plugins included in the GATE distribution
- Other contributed plugins

For more information on how the plugins work, see the online user guide "Developing Language Processing Components with GATE".

To submit a plugin, please contact us via the gate-users mailing
list.

---


## Plugins included in the GATE distribution

- Alignment
- ANNIE
- Annotation\_Merging
- Copy\_Annots\_Between\_Docs
- Gazetteer\_LKB
- Gazetteer\_Ontology\_Based
- GENIA
- Groovy
- Information\_Retrieval
- Inter\_Annotator\_Agreement
- JAPE\_Plus
- Keyphrase\_Extraction\_Algorithm
- Language\_Identification
- Lang\_Arabic
- Lang\_Cebuano
- Lang\_Chinese
- Lang\_Hindi
- Lang\_Romanian
- Learning
- LingPipe
- Machine\_Learning
- Ontology
- Ontology\_BDM\_Computation
- Ontology\_Tools
- OpenNLP
- Parser\_Minipar
- Parser\_RASP
- Parser\_Stanford
- Parser\_SUPPLE
- Schema\_Annotation\_Editor
- Schema\_Tools
- Stemmer\_Snowball
- Tagger\_Abner
- Tagger\_Boilerpipe
- Tagger\_Chemistry
- Tagger\_DateNormalizer
- Tagger\_Framework
- Tagger\_Measurements
- Tagger\_MetaMap
- Tagger\_MutationFinder
- Tagger\_NormaGene
- Tagger\_NP\_Chunking
- Tagger\_Numbers
- Tagger\_OpenCalais
- Tagger\_PennBio
- Teamware\_Tools
- Tools
- UIMA
- Web\_Crawler\_Websphinx
- WordNet

| Alignment | | |
| --- | --- | --- |
| Compound Document | GATE Compound Document. (docs) | gate.compound.impl.CompoundDocumentImpl |
| Compound Document From Xml | GATE Compound Document. (docs) | gate.compound.impl.CompoundDocumentFromXml |
| Compound Document Editor | Editor for compound documents. (docs) | gate.compound.gui.CompoundDocumentEditor |
| GATE Composite document | GATE Composite document. (docs) | gate.composite.impl.CompositeDocumentImpl |
| Switch Member PR | Sets the focus of a compound document to a specified member document. (docs) | gate.compound.impl.SwitchMemberPR |
| Delete Member PR | Deletes one member document from a compound doc. (docs) | gate.compound.impl.DeleteMemberPR |
| Combine Members PR | Combines documents in a composite document. (docs) | gate.composite.impl.CombineMembersPR |
| Segment Processing PR | Processes individual segments as separate documents (docs) | gate.composite.impl.SegmentProcessingPR |
| ExportAlignmentPR | A PR to export alignment information in an xml file. | gate.alignment.ExportAlignmentPR |
| ANNIE | | |
| Annotation Schema | An annotation type and its features. (docs) | gate.creole.AnnotationSchema |
| GATE Unicode Tokeniser | A customisable Unicode tokeniser. (docs) | gate.creole.tokeniser.SimpleTokeniser |
| ANNIE English Tokeniser | A customisable English tokeniser. (docs) | gate.creole.tokeniser.DefaultTokeniser |
| ANNIE Gazetteer | A list lookup component. (docs) | gate.creole.gazetteer.DefaultGazetteer |
| Sharable Gazetteer | A sharable list lookup component. (docs) | gate.creole.gazetteer.SharedDefaultGazetteer |
| Hash Gazetteer | A list lookup component implemented by OntoText Lab. The licence information is also available in licence.ontotext.html in the lib folder of GATE (docs) | com.ontotext.gate.gazetteer.HashGazetteer |
| JAPE Transducer | A module for executing Jape grammars. (docs) | gate.creole.Transducer |
| ANNIE NE Transducer | ANNIE named entity grammar. (docs) | gate.creole.ANNIETransducer |
| ANNIE Sentence Splitter | ANNIE sentence splitter. (docs) | gate.creole.splitter.SentenceSplitter |
| RegEx Sentence Splitter | A sentence splitter based on regular expressions. (docs) | gate.creole.splitter.RegexSentenceSplitter |
| ANNIE POS Tagger | Mark Hepple's Brill-style POS tagger. (docs) | gate.creole.POSTagger |
| ANNIE OrthoMatcher | ANNIE orthographical coreference component. (docs) | gate.creole.orthomatcher.OrthoMatcher |
| ANNIE Pronominal Coreferencer | Pronominal Coreference resolution component. (docs) | gate.creole.coref.Coreferencer |
| ANNIE Nominal Coreferencer | Nominal Coreference resolution component (docs) | gate.creole.coref.NominalCoref |
| Document Reset PR | Document cleaner. (docs) | gate.creole.annotdelete.AnnotationDeletePR |
| Jape Viewer | A JAPE grammar file viewer. (docs) | gate.gui.jape.JapeViewer |
| Gazetteer Editor | Gazetteer viewer and editor. (docs) | gate.gui.GazetteerEditor |
| Annotation\_Merging | | |
| Annotation Merging PR | Merge Annotations from different annotators. (docs) | gate.merger.AnnotationMergingMain |
| Copy\_Annots\_Between\_Docs | | |
| Copy Anns to Another Doc PR | Copy the annotations from one document to another document. (docs) | gate.copyAS2AnoDoc.CopyAS2AnoDocMain |
| Gazetteer\_LKB | | |
| Large KB Gazetteer | KIM KB based alias-lookup commponent (docs) | com.ontotext.kim.gate.KimGazetteer |
| Semantic Enrichment PR | The Semantic Enrichment PR allows adding new data to semantic annotations by querying external RDF (Linked Data) repositories. (docs) | com.ontotext.kim.gate.SesameEnrichment |
| Gazetteer\_Ontology\_Based | | |
| Onto Root Gazetteer | A ontology lookup component (docs) | gate.clone.ql.OntoRootGaz |
| Fake Sentence Splitter | Fake Sentence Splitter is used by Onto Root Gazetteer internally as it creates 'fake' annotation type 'Sentence' without analysing the text by a proper Sentence Splitter. The reason for doing this is enabling the POS Tagger to work properly, as the input text is usually not a proper sentence (i.e. ontology resource name or label). 'Faking' sentence splitting optimises the processing as Onto Root Gazetteer usually does not process internally any multisentence text. | gate.clone.ql.FakeSentenceSplitter |
| GENIA | | |
| GENIA Sentence Splitter | A processing resource that takes document and corpus parameters (docs) | gate.creole.genia.splitter.GENIASentenceSplitter |
| Groovy | | |
| Groovy support for GATE |  | gate.groovy.GroovySupport |
| Groovy scripting PR | Runs a Groovy script as a processing resource (docs) | gate.groovy.ScriptPR |
| Scriptable Controller | A controller whose execution strategy is controlled by a Groovy script (docs) | gate.groovy.ScriptableController |
| Control Script | Editor for the Groovy script controlling a scriptable controller | gate.groovy.gui.ControllerScriptEditor |
| Script Editor | Editor for the Groovy script behind this PR | gate.groovy.gui.ScriptPREditor |
| Information\_Retrieval | | |
| SearchPR | Provides IR functionality. (docs) | gate.creole.ir.SearchPR |
| Search Results | Viewer for IR search results | gate.gui.SearchPRViewer |
| Inter\_Annotator\_Agreement | | |
| IAA Computation PR | Compute inter-annotator agreement (IAA). (docs) | gate.iaaplugin.IaaMain |
| JAPE\_Plus | | |
| JAPE-Plus Viewer | A JAPE grammar file viewer (docs) | gate.gui.jape.plus.Viewer |
| JAPE-Plus Transducer | An optimised, JAPE-compatible transducer. | gate.jape.plus.Transducer |
| Keyphrase\_Extraction\_Algorithm | | |
| KEA Keyphrase Extractor | A Keyphrase Extractor by Eibe Frank. (docs) | gate.creole.kea.Kea |
| KEA Corpus Importer | Imports a KEA-style corpus into GATE | gate.creole.kea.CorpusImporter |
| Language\_Identification | | |
| TextCat Fingerprint Generator | Generate language fingerprints for use with the TextCat Language Indentification PR (docs) | org.knallgrau.utils.textcat.FingerprintGenerator |
| TextCat Language Identification | Recognizes the document language using TextCat (docs) | org.knallgrau.utils.textcat.LanguageIdentifier |
| Lang\_Arabic | | |
| Arabic Tokeniser | A customisable Arabic tokeniser. | arabic.ArabicTokeniser |
| Arabic Gazetteer | A list lookup component. | arabic.ArabicGazetteer |
| Arabic Infered Gazetteer | A list lookup component. | arabic.ArabicInferedGazetteer |
| Arabic Main Grammar | A module for executing Jape grammars | arabic.ArabicTransducer |
| Arabic OrthoMatcher | Arabic Orthomatcher | arabic.ArabicOrthoMatcher |
| Lang\_Cebuano | | |
| Cebuano Tokeniser | A customisable Cebuano tokeniser. | cebuano.CebuanoTokeniser |
| Cebuano Gazetteer | A list lookup component. | cebuano.CebuanoGazetteer |
| Cebuano Gazetteer Tokeniser | A list lookup component. | cebuano.CebuanoGazetteerTokeniser |
| Cebuano Transducer | A module for executing Jape grammars | cebuano.CebuanoTransducer |
| Cebuano Transducer Postprocessor | A module for executing Jape grammars | cebuano.CebuanoTransducerPost |
| Cebuano POS Tagger | Mark Hepple's Brill-style POS tagger, adapted for languages where entries are multiword | cebtag.postag.CebuanoPOSTagger |
| Lang\_Chinese | | |
| Chinese Segmenter PR | Segment the Chinese text into words, based on the PAUM learning algorithm. (docs) | gate.chineseSeg.ChineseSegMain |
| Chinese IE System |  | chinese.ChineseIE |
| Lang\_Hindi | | |
| Hindi Tokeniser | A customisable Hindi tokeniser. | hindi.HindiTokeniser |
| Hindi Gazetteer | A list lookup component. | hindi.HindiGazetteer |
| Hindi Splitter | A Sentence Splitter. | hindi.HindiSplitter |
| Hindi Tokeniser Gazetteer | A list lookup component. | hindi.HindiTokeniserGazetteer |
| Hindi Main Grammar | A module for executing Jape grammars | hindi.HindiTransducer |
| Hindi Tokeniser Postprocessor | A module for executing Jape grammars | hindi.HindiTokeniserPostprocessor |
| Hindi OrthoMatcher | Hindi Orthomatcher | hindi.HindiOrthoMatcher |
| Hindi POS Tagger | Mark Hepple's Brill-style POS tagger, adapted for languages where entries are multiword | cebtag.postag.CebuanoPOSTagger |
| Lang\_Romanian | | |
| Romanian Tokeniser | A customisable Romanian tokeniser. | romanian.RomanianTokeniser |
| Romanian Gazetteer | A list lookup component. | romanian.RomanianGazetteer |
| Romanian Transducer | A module for executing Jape grammars | romanian.RomanianTransducer |
| Learning | | |
| Batch Learning PR | Supports training, application and evaluation of machine learning models for NLP tasks (docs) | gate.learning.LearningAPIMain |
| LingPipe | | |
| LingPipe Tokenizer PR | Provides a LingPipe tokenizer. (docs) | gate.lingpipe.TokenizerPR |
| LingPipe NER PR | LingPipe Named Entity Recognizer (docs) | gate.lingpipe.NamedEntityRecognizerPR |
| LingPipe Language Identifier PR | GATE PR for language identification using LingPipe (docs) | gate.lingpipe.LanguageIdentifierPR |
| LingPipe POS Tagger PR | Provides a LingPipe part of speech tagger. (docs) | gate.lingpipe.POSTaggerPR |
| LingPipe Sentence Splitter PR | Provides an interface to LingPipe sentence splitter API. (docs) | gate.lingpipe.SentenceSplitterPR |
| Machine\_Learning | | |
| Machine Learning PR | Trains a machine learning algorithm from a corpus. For new code, consider using the "learning" plugin instead. (docs) | gate.creole.ml.MachineLearningPR |
| Ontology | | |
| ConnectSesameOntology | Connect to a repository containing and ontology (docs) | gate.creole.ontology.impl.sesame.ConnectSesameOntology |
| CreateSesameOntology | Create a ontology from a Sesame configuration file for a repository (docs) | gate.creole.ontology.impl.sesame.CreateSesameOntology |
| OWLIM Ontology | Ontology created as a temporary OWLIM3 in-memory repository (docs) | gate.creole.ontology.impl.sesame.OWLIMOntology |
| OWLIM Ontology DEPRECATED | Ontology created as a temporary OWLIM3 in-memory repository, for backwards compatibility only (docs) | gate.creole.ontology.owlim.OWLIMOntologyLR |
| Ontology\_BDM\_Computation | | |
| BDM Computation PR | Compute BDM score for each pair of concepts in the given ontology. (docs) | gate.bdmComp.BDMCompMain |
| Ontology\_Tools | | |
| OntoGazetteer | A list lookup component based on mapping between ontology classes and gazetteer lists. (docs) | gate.creole.gazetteer.OntoGazetteerImpl |
| GATE Ontology Editor | Ontology editing tool. (docs) | gate.gui.ontology.OntologyEditor |
| OAT | Ontology Annotation Tool. (docs) | gate.creole.ontology.ocat.OntologyViewer |
| RAT-C | Relation Annotation Tool Class view. (docs) | gate.gui.docview.OntologyClassView |
| RAT-I | Relation Annotation Tool Instance view. (docs) | gate.gui.docview.OntologyInstanceView |
| GAZE | Gazetteer viewer and editor (docs) | com.ontotext.gate.vr.Gaze |
| OpenNLP | | |
| OpenNlpSentenceSplit | Gate wrapper of the OpenNlp Sentence Splitter. (docs) | gate.opennlp.OpenNlpSentenceSplit |
| OpenNlpTokenizer | Implementation of the OpenNlp Token Splitter. (docs) | gate.opennlp.OpenNlpTokenizer |
| OpenNlpPOS | Implementation of the OpenNlp POS Tagger. (docs) | gate.opennlp.OpenNlpPOS |
| OpenNlpChunker | Implementation of the OpenNlp Chunker. (docs) | gate.opennlp.OpenNlpChunker |
| OpenNlpNameFinder | Implementation of the OpenNlp Name Finder. (docs) | gate.opennlp.OpenNLPNameFin |
| Parser\_Minipar | | |
| Minipar Wrapper | MiniPar is a shallow parser. It determines the dependency relationships between the words of a sentence. (docs) | minipar.Minipar |
| Parser\_RASP | | |
| RASP2 Tokenizer | RASP2 Tokenizer. Faster than the original GATE component but generates Tokens which have only a 'string' feature. Requires annotations of type Sentence. See RASP package for platform restrictions. (docs) | com.digitalpebble.rasp2.token.RASPTokenizer |
| RASP POS Converter | Converts from PennTreebank POS tags to the C2 tagset used by RASP. Generates annotations of type MorphObj which hold the tag and lemma (docs) | com.digitalpebble.rasp2.tagger.C2Transducer |
| RASP2 POS Tagger | RASP part-of-speech tagger, creating WordForm annotations (docs) | com.digitalpebble.rasp2.tagger.PosTagger |
| RASP2 Morphological Analyser | RASP morphological analyser, which adds lemma and suffix to the WordForm annotations produced by the RASP POS tagger (or the ANNIE POS tagger plus the RASP converter) (docs) | com.digitalpebble.rasp2.morph.MorphoAnnotator |
| RASP2 Parser | RASP dependency parser (docs) | com.digitalpebble.rasp2.parser.ParserAnnotator |
| Parser\_Stanford | | |
| StanfordParser | Stanford parser wrapper (docs) | gate.stanford.Parser |
| English Dependency Parser |  | gate.stanford.apps.EnglishDependencies |
| English POS Tagger and Dependency Parser |  | gate.stanford.apps.EnglishPOSDependencies |
| Parser\_SUPPLE | | |
| SUPPLE Parser | SUPPLE bottom-up chart parser. (docs) | shef.nlp.supple.SUPPLE |
| Schema\_Annotation\_Editor | | |
| Schema Annotations Editor | An annotation editor restricted by schemas. (docs) | gate.gui.annedit.SchemaAnnotationEditor |
| Schema\_Tools | | |
| Annotation Schema | An annotation type and its features. (docs) | gate.creole.AnnotationSchema |
| Schema Enforcer | Produces an annotation set whose content is restricted by the specified set of schemas (docs) | gate.creole.schema.SchemaEnforcer |
| Simple Schema Viewer | A Simple Annotation Schema Viewer | gate.gui.schema.SimpleSchemaViewer |
| Stemmer\_Snowball | | |
| Stemmer PR | Wrapper for the Snowball stemmer. (docs) | stemmer.SnowballStemmer |
| Tagger\_Abner | | |
| ABNER Tagger | GATE wrapper over ABNER (docs) | gate.abner.AbnerTagger |
| Tagger\_Boilerpipe | | |
| Boilerpipe Content Detection | Uses boilerpipe to determine which sections of a document are interesting content and which are just boilerplate (docs) | gate.creole.boilerpipe.BoilerPipe |
| Tagger\_Chemistry | | |
| Chemistry Tagger | A tagger for chemical names. (docs) | mark.chemistry.Tagger |
| Tagger\_DateNormalizer | | |
| Date Normalizer | provides normalized values for all known dates | gate.creole.dates.DateNormalizer |
| Tagger\_Framework | | |
| GenericTagger | The Generic Tagger is Generic! (docs) | gate.taggerframework.GenericTagger |
| Tagger\_Measurements | | |
| ANNIE+Measurements |  | gate.creole.measurements.ANNIEMeasurements |
| Measurement Tagger | A measurement tagger based upon GNU Units | gate.creole.measurements.MeasurementsTagger |
| Tagger\_MetaMap | | |
| MetaMap Annotator | This plugin uses the MetaMap Java API to send GATE document content to MetaMap skrmedpostctl server and PrologBeans mmserver instances running on the given machine/port (docs) | gate.metamap.MetaMapPR |
| Tagger\_MutationFinder | | |
| MutationFinder | GATE MutationFinder Wrapper (docs) | gate.creole.mutationfinder.MutationFinderPR |
| Tagger\_NormaGene | | |
| NormaGene Tagger | A processing resource that takes document and corpus parameters | gate.creole.normagene.NormaGene |
| Tagger\_NP\_Chunking | | |
| Noun Phrase Chunker | Implementation of the Ramshaw and Marcus base noun phrase chunker (docs) | mark.chunking.GATEWrapper |
| Tagger\_Numbers | | |
| Numbers Tagger | Finds numbers in (both words and digits) and annotates them with their numeric value (docs) | gate.creole.numbers.NumbersTagger |
| Roman Numerals Tagger | Finds and annotates Roman numerals (docs) | gate.creole.numbers.RomanNumeralsTagger |
| Tagger\_OpenCalais | | |
| OpenCalais Tagger | An OpenCalais based semantic annotator (docs) | gate.opencalais.OpenCalais |
| Tagger\_PennBio | | |
| Penn BioTagger: Genes | A processing resource that takes document and corpus parameters (docs) | gate.creole.pennbio.GeneTagger |
| Penn BioTagger: Malignancy | A processing resource that takes document and corpus parameters (docs) | gate.creole.pennbio.MalignancyTagger |
| Penn BioTokenizer | A processing resource that takes document and corpus parameters (docs) | gate.creole.pennbio.Tokenizer |
| Penn BioTagger: Variation | A processing resource that takes document and corpus parameters (docs) | gate.creole.pennbio.VariationTagger |
| Teamware\_Tools | | |
| QA Summariser for Teamware | The Quality Assurance PR for teamware (docs) | gate.qa.QAForTeamwarePR |
| Tools | | |
| Gazetteer List Collector | Gazetteer lists collector. (docs) | gate.creole.GazetteerListsCollector |
| ANNIE VP Chunker | ANNIE VP Chunker component. (docs) | gate.creole.VPChunker |
| Annotation Set Transfer | Annotation set transfer component. (docs) | gate.creole.annotransfer.AnnotationSetTransfer |
| Flexible Exporter | Exports a document with GATE annotations to its original format. (docs) | gate.creole.dumpingPR.DumpingPR |
| GATE Morphological analyser | Morphological Analyzer for the English Language. (docs) | gate.creole.morph.Morph |
| Flexible Gazetteer | A more flexible list lookup component. (docs) | gate.creole.gazetteer.FlexibleGazetteer |
| Syntax tree viewer | Viewer for syntax trees generated by a parser. (docs) | gate.gui.SyntaxTreeViewer |
| Quality Assurance PR | The Quality Assurance PR provides a functionality of the Corpus QA Tool in GATE Developer | gate.qa.QualityAssurancePR |
| UIMA | | |
| UIMA Analysis Engine | Wrapper for a Text Analysis Engine from UIMA. (docs) | gate.uima.AnalysisEnginePR |
| Web\_Crawler\_Websphinx | | |
| Crawler PR | GATE implementation of the Websphinx crawling API (docs) | crawl.CrawlPR |
| WordNet | | |
| WordNet 1.6 | Princeton WordNet 1.6. (docs) | gate.wordnet.IndexFileWordNetImpl |
| WordNet | WordNet (docs) | gate.wordnet.JWNLWordNetImpl |
| WordNet Viewer | WordNet viewer | gate.gui.wordnet.WordNetViewer |

---


## Other contributed plugins

- OrganismTagger
- Multi-lingual Noun Phrase Extractor (MuNPEx)
- Durm German lemmatizer
- XCES tools
- *Sen* wrapper (Japanese morphological analyser)
- Russian morph tagger
- String Annotation
- GATE Application Documentation
- VirtualCorpus - Directory- and JDBC Corpus LR
- BWP Gazetteer
- Apolda
- Reported Speech Tagger
- Keyphrase extraction module from SmILE

| OrganismTagger website download | | |
| --- | --- | --- |
| OrganismTagger | The OrganismTagger is a hybrid rule-based/machine-learning system that extracts organism mentions from the biomedical literature, normalizes them to their scientific name, and provides grounding to the NCBI Taxonomy database.  | |  |
| Multi-lingual Noun Phrase Extractor (MuNPEx) website download | | |
| Munpex | MuNPEx is a multi-lingual noun phrase (NP) extraction component developed for the GATE architecture, implemented in JAPE. It currently supports English, German, French, and Spanish (in beta). | en-np\_main.jape |
| Durm German lemmatizer website download | | |
| Durm German lemmatizer | The Durm German Lemmatization System consists of a number of GATE components and resources that perform morphological analysis and lemmatization for German nouns. |  |
| XCES tools website download | | |
| *Tools to handle documents conforming to the XML Corpus Encoding Standard (XCES) format, used by the American National Corpus. XCES is a way of encoding texts with standoff markup in XML.* | | |
| ANC Document | An XCES document. Allows loading of the document text, plus some or all of the sets of standoff markup associated with the document. | org.xces.gate.XCESDocument |
| ANC Load Standoff | Loads standoff annotations into an existing document. | org.xces.creole.LoadStandoff |
| ANC Save Content | Saves just the text content of a document to a file. This will work for any document - it is not specific to ANC/XCES documents. | org.xces.creole.SaveContent |
| ANC Save Standoff | Saves annotations from a Document to an XCES-compliant standoff markup file. | org.xces.creole.SaveStandoff |
| *Sen* wrapper (Japanese morphological analyser) website (in Japanese) | | |
| *Sen is a morphological analyser for Japanese. This is a wrapper to allow it to be used from GATE, you must also install sen itself. See the documentation (in Japanese) for details.* | | |
| Sen Wrapper | Morphological analyser for Japanese | jp.co.ditlab.jgate.SenWrapper |
| Russian morph tagger website download | | |
| *Provides GATE wrapper for mystem russian morphological parser. Allows to execute native analyzer, parse output, and assign morpho features as GATE annotations for the document.* | | |
| Russian MorphTagger | MorphTagger for russian language, based on MyStem Yandex' parser | ru.itbrains.gate.morph.MorphTagger |
| String Annotation Plugin  website download | | |
| *Processing resources for directly annotating the string content of a document.* | | |
| Extended List Gazetteer | Extended version of the GATE Default List Gazetteer. In addition to the features of the original, built-in version of the List Gazetteer, this version provides features for more powerful matching of partial words and annotating prefixes and suffixes as well as more versatile handling of word boundaries and whitespace. | at.ofai.gate.extendedgazetteer.ExtendedGazetteer |
| Simple Regexp Annotator | Use rules based on Java regular expressions to annotate the document content. | at.ofai.gate.regexpannotator.SimpleRegexpAnnotator |
| AppDoc — GATE Application Documentation  website download | | |
| *A plugin that generations documentation from your application/pipeline/gapp files in various formats. In addition it provides new visual resources in the GATE Developer GUI to add author, version and documentation comments to pipelines and processing resources.* | | |
| AppDoc | Visual resource for adding author/version/comment to pipelines and processing resources | at.ofai.gate.appdoc.AppDoc |
| AppDocGen | Visual resource for selecting a documentation template and generating documentation files | at.ofai.gate.appdoc.AppDocGen |
| VirtualCorpus — Directory- and JDBC Corpus LRs  website download | | |
| *A plugin that provides two new corpus language resources, DirectoryCorpus for directly using files in a directory through a corpus LR and JDBCCorpus for directly using documents stored in a field of a JDBC database table.* | | |
| DirectoryCorpus | Language resource for accessing GATE XML files in a directory directly via a corpus resource | at.ofai.gate.virtualcorpus.DirectoryCorpus |
| JDBCCorpus | Language resource for accessing GATE XML documents stored in a field of a JDBC database table directly via a corpus resource | at.ofai.gate.virtualcorpus.JDBCCorpus |
| BWP Gazetteer  website download | | |
| *This plugin provides an approximate gazetteer for GATE, based on Levenshtein's Edit Distance for strings. Its goal is to handle texts with noise and errors, in which GATE's default gazetteers may have difficulties.* | | |
| BWP Gazetteer | Extended version of the transducer-based List Gazetteer |  |
| Apolda  website download | | |
| *Annotates documents like a gazetteer, but takes the terms from OWL annotation properties in an ontology, rather than from a separate list of terms.* | | |
| Apolda Ontology Annotator | Ontology-based lookup taking terms from properties in the ontology. | telin.Apolda |
| Reported Speech Tagger  website download | | |
| *Automatically detects and tags reported speech constructs, in particular the source, reporting verb and content.* | | |
| Reporting Verb marker | JAPE transducer which tags reporting verbs |  |
| Reported Speech finder | JAPE transducer which tags reported speech |  |
| Keyphrase Extraction Module  website download | | |
| *Keyphrase extraction and language identification.* | | |
| FrequencyAnalyser |  |  |
| KeywordAnalyser |  |  |
| LanguageIdentification | identifies the language of a document using character n-grams |  |
| POSTagMapper |  |  |
| SimpleNounChunking |  |  |
| StopwordMarker |  |  |

---

| gate home |

|  |  |
| --- | --- |
|  |  |
